# Early Bifrontal Brain Injury: Disturbances in Cognitive Function Development

**DOI:** 10.1155/2010/765780

**Published:** 2010-09-26

**Authors:** Christine Bonnier, Aurélie Costet, Ghassan Hmaimess, Corinne Catale, Christelle Maillart, Patricia Marique

**Affiliations:** ^1^Service de Neurologie Pédiatrique, Centre Neurologique William Lennox, Cliniques Saint-Luc, Université catholique de Louvain, Avenue Hippocrate, 10/1067, Ottignies-Louvain-la-Neuve, 1200 Bruxelles, Belgium; ^2^Département des Sciences Cognitives, Faculté de Psychologie et des Sciences de l'Education, Université de Liège, 4000 Liège, Belgium

## Abstract

We describe six psychomotor, language, and neuropsychological sequential developmental evaluations in a boy who sustained a severe bifrontal traumatic brain injury (TBI) at 19 months of age. Visuospatial, drawing, and writing skills failed to develop normally. Gradually increasing difficulties were noted in language leading to reading and spontaneous speech difficulties. The last two evaluations showed executive deficits in inhibition, flexibility, and working memory. Those executive abnormalities seemed to be involved in the other impairments. In conclusion, early frontal brain injury disorganizes the development of cognitive functions, and interactions exist between executive function and other cognitive functions during development.

## 1. Introduction

Traumatic brain injury (TBI) sustained at a very young age is associated with high rates of long-term morbidity and mortality. The aetiology and pathophysiology of head injuries in children younger than 4 years of age differ from those in older children. Although the causes of TBI in children under 4 include falls, as well as traffic accidents as passengers [1, 2], whose prevalence increases from infancy (23% of all TBIs) to adolescence (82%) [3], the proportion of nonaccidental head injuries (NAHI) is very high, between 20% and 75% [4–7]. Nonaccidental head injury is associated with worse outcomes than noninflicted TBI [8].

The first postnatal years are characterized by an extremely fast rate of brain growth, which involves numerous processes such as dendritogenesis, axogenesis, synaptogenesis and synaptic stabilisation, gliogenesis, and myelination [9–14]. TBI in very young children is associated with greater severity of impairments in language, attention, fine motor speed, tactile recognition, and visuospatial functions, compared to older children [15–19]. The influence of age at injury on the long-term cognitive outcomes of TBI interacts with a number of others factors, including time since injury, vulnerability of specific functions, and site and extent of brain damage [20].

Differences in outcomes according to which cerebral lobe is involved, and more specifically the impact of frontal lobe damage, have been investigated in several series of school-aged children. Several studies assessed the influence of frontal lobe involvement on cognitive functions, including intellectual and executive functions [21], emotional aspects of narratives [22], and verbal memory [23]. Greater impairment of these functions was associated with younger age at injury and with involvement of one or both frontal lobes. In one study, however, frontal lobe involvement was not associated with behavioural outcomes or adaptive functioning [24]. Moreover, frontal lesion size was not associated with measures of executive function in a study of children aged 7 to 15 years [25]. Differential effects of right versus left frontal lobe injury at 5 to 15 years of age were looked for in a longitudinal study [26]. The results showed an interaction between age and TBI severity: thus, word fluency recovery was slower after severe TBI in younger children than after severe TBI in older children or mild TBI in younger children. In addition, involvement of the left frontal lobe was associated with worse word-fluency performance in older children, compared to younger children and to involvement of the right frontal lobe. This result may be ascribable to a greater functional commitment of the left frontal lobe to expressive language and word fluency in older than in younger children and/or to the combined influence of expressive language deficiency and executive function impairment.

Little is known about the impact of frontal lobe involvement on outcomes after TBI in very young children. A single case-series of children with frontal lobe injury included patients younger than 6 years at injury [21]. General intelligence was assessed using the WISC, and four tests of executive functions were performed. The results showed that frontal lobe injury impaired the development of both executive skills and general intelligence. Younger age at injury was associated with a poorer rate of performance. Furthermore, executive function impairment was closely linked to intellectual function [21].

A number of case-reports have supplied information on adulthood outcomes after frontal lobe injury sustained in early childhood [27–30]. Overall, these reports indicate that early prefrontal injury can lead to impairments not only in executive functions (e.g., planning or decision-making), but also in social behaviour. Two young adults with a history of prefrontal injury before 16 months of age had impaired decision making, behavioural dyscontrol, social defects, and abnormal emotion [31] contrasting with normal performance on tests for intellect, memory, language, and perception. In a patient who sustained extensive damage to the right dorsolateral prefrontal cortex at age 7, evaluations 4 and 8 years later showed significant improvements in many cognitive areas including working memory and several attentional and executive tasks (such as design fluency and planning), with no evidence of social impairment, abnormal personality, or moral disturbances [32]. However, attentional difficulties and impulsive responding were noted. We are not aware of other patients in whom longitudinal data were collected after early TBI. Because most of the patients were evaluated only in adulthood, it is unclear whether the cognitive or behavioural deficits were direct consequences of the frontal lobe damage. Repeated testing over time is required to study the developmental impact of early frontal-lobe injury and to look for interactions with other factors.

Here, we report on a patient who sustained bilateral frontal lobe damage at 19 months of age during a domestic accident and who was subsequently evaluated six times between the ages of 3 years 9 months and 9 years 10 months. The development of psychomotor, language, and executive functions in this patient is described below.

## 2. Method

### 2.1. Case History

The patient was a boy born to Moroccan parents living in the French-speaking part of Belgium. Delivery was normal. He learned to walk at 13 months. Both French and Arabic were spoken at home, and his language development was normal.

**Figure 1:**
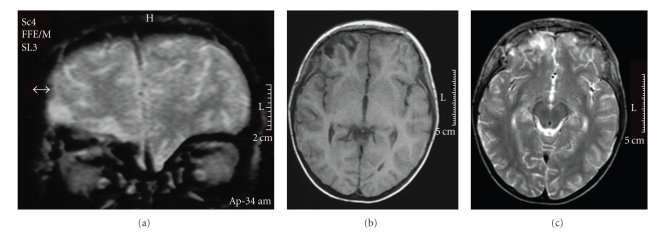
Magnetic Resonance Imaging (MRI) performed 2 weeks after trauma: FFE-coronal (a), T1 weighed (b), and T2-axial (c) sections showed bilateral (right > left) basi-frontal lesions. Right insular lesion is not shown.

**Figure 2:**
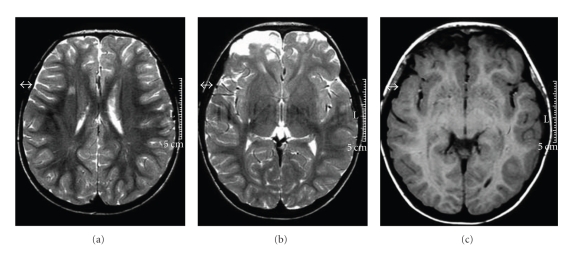
A control MRI was performed 2 years after trauma: FLAIR section (a) showed stable white matter lesions (right fronto-parietal and left posterior-parietal), T2-weighed (b) and T1-axial (c) sections showed worsening in bi-frontal atrophy.

At 19 months of age, he fell from a height of six meters. He did not lose consciousness. He was taken immediately to the emergency room, where the physical examination showed a deep wound in the forehead, bilateral epistaxis, periorbital hematoma, and palpebral oedema. He was irritable and a language deficit was noted. His deep tendon reflexes and plantar reflexes were normal but he had mild paresis of the left lower limb. Computed tomography (CT) of the brain showed multiple fractures of the frontal bone with displaced fragments; fractures of the ethmoidal bone, right supraorbital area, and right nasal bone; and brain oedema. Surgery of the displaced frontal bone fracture was performed 2 days after the fall. The postoperative course was favourable, with gradual improvement of the paresis of the left lower limb. Two weeks later, magnetic resonance imaging (MRI) disclosed bilateral high signal from the base of the frontal cortex, as well as a right anterior insular lesion. Two years later, a control MRI showed stability in white matter lesions, but atrophy of bifrontal cortex was more severe (Figures 1 and 2). Electroencephalography showed asymmetrical activity that was better on the left side. Visual, auditory, and sensory potentials were normal. He recovered the ability to walk 2 months after the fall. His language, however, remained poor. Finally, late posttraumatic epilepsy characterized by partial seizures developed 2 years after the injury. Carbamazepine was effective in stabilizing the seizures. He received motor and speech rehabilitation therapy outside our unit and was enrolled in a special-education program at 7 years of age.

He was first evaluated at our clinical unit when he was 3 years 9 months old. Subsequently, we re-evaluated him once a year, for a total of six evaluations; thus, at the last evaluation, he was 9 years 10 months old. His cognitive development was assessed using measures of motor function, language, and neuropsychological status.

### 2.2. Behaviour as Observed by the Parents, Teachers, and Health Professionals

His parents were attentive observers, who noticed changes over the years. Immediately after the injury, they detected no behavioural or emotional changes, except regarding language. However, 2 months later, his parents described him as “hyperactive” at home, moving around continuously and unable to remain still or to play the same game for more than a few minutes.

He started to attend nursery school 18 months after the injury, when he demonstrated difficulties relating to his peers. He spoke only about five words and was extremely restless. Psychomotor disturbances emerged at that time: thus, although he was then 3 years old he was unable to distinguish a circle from a square, to pedal on a tricycle, or to put his finger on his mouth or nose. Consequently, psychomotor and speech rehabilitation therapy was started. About 6 months later (when he was 3 years 6 months, 2 years after the injury), he had attentional deficits, delayed language development with a combination of vocabulary deficiency and receptive language impairment, and delayed motor development. He experienced considerable difficulty relating to other children. French was chosen as his main language, as bilingualism was felt to be too complicated for him.

Three years after the injury (when he was 4 years 10 months old), he was less hyperactive but exhibited major attention deficiency, particularly in his class group, where he was easily distractible and seemed not to hear others. One year later (when he was 5 years 9 months old, 4 years after the injury), he was described as very impulsive, with deficits in expressive language and in psychomotor skills. He therefore repeated his third year of nursery school. When he was 7 years old, his persistent difficulties with attention and language led to his enrolment in a special-education program.

During the last two evaluations at our clinical unit, he seemed excessively obedient and polite: for example, he did not ask for food when he was hungry. On the other hand, he occasionally exhibited symptoms of hyperactivity, running and jumping during games that did not require physical activity. At school, he was described as the perfect student, being interested in everything, very polite, and reserved. His parents, however, reported difficulty concentrating on his homework. The last evaluation was performed when the parents asked that he be returned to the mainstream school system, as they felt he no longer required special education. No parental scales were used to confirm these observations of family.

### 2.3. Tests

Cognitive development was assessed using measures of (a) general intellectual abilities, (b) attentional and executive functions, (c) working memory, (d) language, and (e) psychomotor skills. 

#### 2.3.1. General Intellectual Abilities

We chose among the following scales based on age at evaluation: Wechsler Intelligence Scale for Pre-school Children—Revised [33], Wechsler Intelligence Scale for Children—Third Edition [34], and Wechsler Intelligence Scale for Children—Fourth Edition [35].

#### 2.3.2. Attentional and Executive Functions

Few tools are available for measuring these functions in young children. An increasing number of tests were performed from one evaluation to the next, as the patient advanced in age.

**Visual Selective Attention**

The following tests were used.

- Crossing tasks from the NEPSY [36]: the child is instructed to select only the items (part 1: cats; part 2: faces) that match the target stimuli on pages containing both targets and distractors. The number of correctly identified targets (maximum 20 cats and faces) and the completion time (maximum 180 seconds) are scored.
- The visual selective attention task on the TAP (Test Battery for Attentional Performance [37]): crosses appear in a random configuration on a 4×4 matrix, and the child is asked to press a key when four of the crosses form a square. Reaction times, missed responses, and unwarranted responses are recorded.

**Divided Attention**

A dual task from the TAP [37] was used. The child must simultaneously perform visual and auditory selective attention tasks. The visual attention task was the same as above.

**Sustained Attention**

The 10-minute Zazzo Cancellation Task was used. The child is asked to cross out as fast as possible two kinds of target signs on sheets that also contain distractor signs, for 10 minutes. Time to completion and accuracy are recorded.

**Inhibition**

Both cognitive and behavioural inhibitions were measured, using the tests listed below.

- Statue test from the NEPSY [36]: the child is asked to remain standing, pretending to hold a flag, with the eyes closed and no vocalizations, for 75 seconds.
- Knock-and-tap test from the NEPSY [36]: the child is asked to knock on the table with the knuckles when the examiner taps the table with the palm and vice versa. A total of 15 trials are performed in pseudorandom order.
- Go/No-Go task from the TAP [37] and KITAP [38]: the child must press a key as fast as possible when a target (a cross in the TAP or a bat in the KITAP) appears on the screen but not when other images resembling the target are displayed.
- The Interference Fruit Task [39], based on the Stroop Word Test and adapted from the fruit task developed by Archibald and Kerns [40]. This task includes three conditions that can be performed by young children who have not yet learned to read. Contrary to the fruit task developed by Archibald and Kerns [40], unlimited time is allowed for naming the colours of the rectangles. Furthermore, we included only three common fruits (banana, pear, and strawberry), whose colours (yellow, green, and red) are well known by young children. In the first condition, the child is asked to name colours of 45 rectangles as quickly as possible, to provide a measure of processing speed. In the interference condition, the child must say the correct colours of 45 incorrectly coloured pictures of fruits, (e.g., “yellow” when shown a picture of a green banana). Total completion time and errors are scored for both conditions.
- Incompatibility (TAP [37]). In this task, arrows pointing to the left or right are shown on the left or right side of a fixed point. The child must press a key on the left or right, depending on the direction of the arrow, irrespective of the location of the arrow relative to the point. When the side and direction of the arrow are the same (i.e., leftward-pointing arrow to the left of the point), the condition is classified as compatible; otherwise, the condition is classified as incompatible. Reaction times and errors in compatible and incompatible conditions are scored.

**Cognitive Flexibility**

Spontaneous and reactive flexibility [41] were assessed. To assess spontaneous flexibility, we used 1-minute fluency tasks. Verbal fluency was measured based on the names of animals, beverages, and foods, using normative data from the NEPSY. Visuospatial fluency was evaluated using the design fluency task from the NEPSY. For all fluency tasks, the total number of correct responses was determined. To evaluate reactive flexibility, we chose the flexibility task from the TAP [37], in which a letter and a number are displayed side by side on the screen. The child is asked to react alternatively to the letter and to the number by pressing the corresponding key. Mean reaction times and errors are recorded.

**Planning and Organization**

The Tower of London test was used. Number of models built successfully at the first attempt, total number of trials needed to build the 12 models, planning times, and execution times were recorded.

#### 2.3.3. Working Memory

Both verbal and visuospatial tasks requiring working memory were evaluated. The verbal tasks focused on the temporary retention of words or numbers. The visuospatial tasks assessed the temporary retention of sequential or simultaneous images. Working-memory tests included the hand movements and spatial memory tasks from the KABC scale [42].

#### 2.3.4. Language

**Expressive Language**

**Speech Praxis**

It was evaluated using the praxis tests from the BEPL [43] until the patient was 5 years old and the Henin praxis test [44] thereafter. The child was asked to imitate movements made by the examiner, such as pulling out the tongue, puffing out the cheeks, or giving a kiss.

**Semantic Fluency**

We used the MSCA subtest [45], in which the child is asked to name as many foods, animals, clothing items, and methods of transportation as possible, in 20 seconds for each category.

**Phonetic Fluency**

We used a test from the L2MA [46], in which the child must say as many words as possible that start with the phonemes /p/ and /f/, in 1 minute for each category.

**Phonology**

We used the BEPL phonology subtests (PHO1 & PHO2 [43]) until the patient was 6 years old and two subtests from the NEEL (monosyllabic and polysyllabic words [47]) thereafter. In these subtests, the patient is asked to name pictures. Errors in pronunciation are recorded, and the ratio of correctly named pictures over the total number of pictures is determined.

**Vocabulary**

We used the “VOC” subtest from the NEEL [47] and the “vocabulary” (denomination) subtest of the L2MA, in which the child is asked to name pictures. Each correct name is counted, irrespective of whether pronunciation is correct.

**Repetition**

We first used the BEPL [43], in which the child repeats syllables and sentences (subtests Art. & Rph.). After the patient reached 6 years of age, we tested the repetition of syllables and sentences using the NEEL (monosyllabic and polysyllabic words, syntax, and number of words) and the EEL (subtests Rep, PH1 & PH2 [48]). Repetition of nonwords was evaluated using the BELEC [49].

**Morphosyntax**

Two tests were used: the TCG [50], in which the patient is asked to complete a sentence started by the examiner; and TVAP definitions [51, 52], in which the patient must define words said by the examiner.

**Receptive Language**

**Auditory Discrimination**

We used the GNO subtest from the BEPL [43] until the patient was 6 years old. Subsequently, we used the EDP 4–8-year test [53], which requires the child to point to a picture corresponding to a word said by the examiner (e.g., “Show me the hat”).

**Word Comprehension**

The TVAP 3–5 and 5–8-year test [51, 52], as well as the EVIP (Peabody [54]), were used. In these tests, the child points to pictures corresponding to words said by the examiner.

**Sentence Comprehension**

It was evaluated only after 6 years of age, using the O-52 [55] and ECOSSE [56] tests. The child must point to pictures corresponding to sentences said by the examiner (e.g., “Show me the picture where the cat is behind the tree”).

**The Reynell Developmental Language Scales [57]**

They were used until the patient was 6 years of age. These scales assess both receptive and expressive language in children between 1 and 6 years of age.

**Metaphonology**

When the patient was 8 years old, we used three subtests from the BELEC [49] to evaluate the abilities required for written language: syllabic reversal (e.g., if the examiner said “pato”, the child had to say “topa”), phonemic reversal (e.g., if the examiner said “il”, the child had to say “li”), and consonant subtraction (e.g., if the examiner said the nonword “fepa”, the child had to say “epa”).

#### 2.3.5. Psychomotor Skills

Psychomotor development was assessed using the Oseretsky Test [58], which provides a motor quotient. The average motor quotient in the general population is 100 and the standard deviation is 15.

## 3. Results

### 3.1. General Intellectual Development

**Figure 3:**
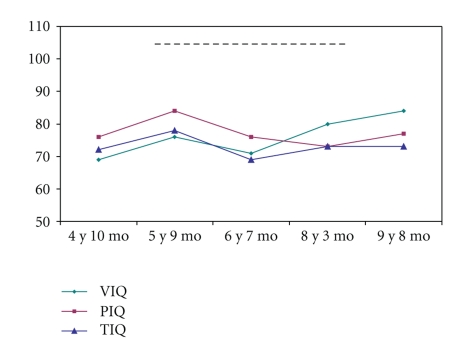
Intellectual evaluation. VIQ: verbal intellectual quotient; PIQ: performance intellectual quotient; TIQ: full-scale intellectual quotient.

The test scores are recapitulated in Table 1 and Figure 3. General intellect was first evaluated when the patient was 4 years 10 months old, using the WPPSI-R. The full-scale IQ was borderline normal (see Table 1). The second evaluation at 5 years 9 months of age showed only a small improvement. The third evaluation was done using the WISC-III at 6 years 7 months of age and yielded results similar to those of the first evaluation using the WPPSI-R. WISC-III results at 8 years 3 months indicated improvements in verbal skills, suggesting a beneficial effect of the special-education program started at 7 years of age. In contrast, nonverbal performances did not improve, and the score on the picture arrangement subtest deteriorated. The last evaluation was done using the WISC-IV at 9 years 8 months and showed results similar to those of the previous WISC-III. Overall, these data indicate steady progress with persistence over time of the same degree of developmental delay.

### 3.2. Developmental Changes in Attentional Function

The first two evaluations performed at 3 years 9 months and 4 years 10 months were somewhat crude given the young age of the patient. The results indicated a deficiency in visual selective attention during NEPSY tasks (bunnies and cats crossing task).

Subsequent assessments were more sophisticated. They were performed at 6 years 7 months, 8 years 3 months, and 9 years 8 months of age (see Table 2). The results showed a persistent deficiency in visual selective attention despite a possible learning effect due to the repeated administration of the same tests. Deficiencies in divided attention and sustained attention were noted at 8 years at age but not at the following evaluation 1 year later.

### 3.3. Developmental Changes in Executive Function

The first evaluations consisted only in the NEPSY statue test, given the young age of the patient (3 years 9 months, 4 years 10 months, and 5 years 9 months). Although the parents reported hyperactivity, the results of the first two evaluations was within the normal range. The third evaluation supported the parents' report of restlessness, as the raw score remained unchanged, at 11. However, a more sophisticated evaluation 1 year later (at 6 years 7 months of age) was within the normal range, with no restlessness, despite reports of inattention at home and at school.

Difficulty with inhibition was noted at the last two evaluations performed when the patient was 8 years 3 months and 9 years 8 months of age (see Table 3). Flexibility was impaired at the first of these two evaluations but was within the normal range the following year. However, perseverative behaviour was noted on several tasks at the last evaluation. For example, during the direct-order digit-span test, the sequence “3417” became “3457” and during the reverse-order test “7296” became “6789”. Similarly, on the Code subtest of the WISC-IV, the symbol ⌉ became the number 7.

Planning and organization were within the normal range at 8 years 3 months of age. The last evaluation (9 years 8 months) showed excessively long planning and execution times on the Tower of London test, although the quality of the response was very good, suggesting that the patient took care to comply with the instruction to make as few mistakes as possible.

### 3.4. Developmental Changes in Working Memory Processes

**Figure 4:**
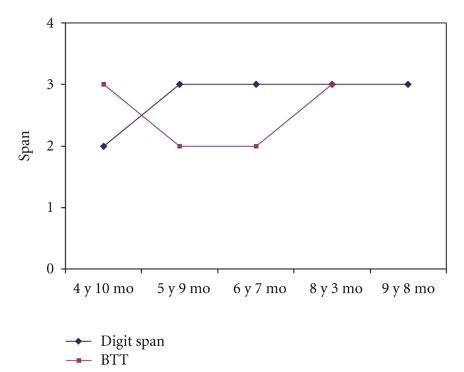
Time-course of verbal and visual spans in working memory.

The serial evaluations showed no progress in working memory for those tests where there is no learning effect (Digit span, Block Tapping Test). Tests from the K.ABC were impaired at the first evaluation and became normal subsequently (see Table 4 and Figure 4). At the last evaluation (9 years 8 months), storage during the phonological loop and visuospatial sketch pad tasks was insufficient overall.

### 3.5. Development of Psychomotor Skills

**Figure 5:**
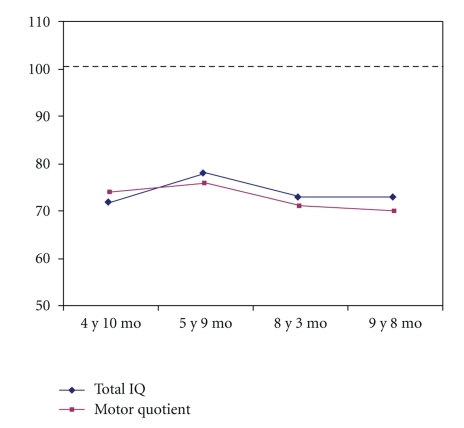
Time-course of the motor quotient from the Oseretsky test and total IQ from the intellectual evaluation (both tests have an average of 100 and a standard deviation of 15 in the general population).

Motor quotients were consistent with the total IQs from the general intellectual evaluations (see Figure 5). At the first evaluation at 3 years 9 months of age, the motor quotient was 73. Despite regular motor rehabilitation therapy (two sessions per week throughout follow-up), his performance remained stable, with motor quotients of 74 at 4 years 10 months and 76 at 5 years 9 months of age (see Table 5). Performance was poorest on balance, speed, simultaneous movements, perceptive graphic organization, and constructive praxis. At the last two evaluations, the motor quotient was still in the borderline range (71 at 8 years 3 months and 70 at 9 years 8 months) and he had persistent difficulties with balance, speed, simultaneous movements, handwriting, and constructive praxis.

### 3.6. Evaluation of Language Development

At the first evaluation, praxis performance and auditory processing were within the normal range; whereas deficiencies were noted in phonology, morphosyntax, and lexical processing (during both expression and comprehension). Praxis remained good at the following evaluations.

The evaluations of expressive language showed weaknesses or deficiencies in phonology until 8 years of age (see Tables 6 and 7). Reduction of multiple consonants was the main difficulty. However, repetition of nonwords and polysyllabic words were within the normal range. Phonology test performance was normal at the last evaluation. Vocabulary was deficient until 6 years of age and improved thereafter, being weak at 8 years and normal at the last evaluation 1 year later. The lexical stock was limited but accessible, with no missing words and good semantic fluency. The bilingual environment in which the patient lived should be borne in mind when interpreting the test results. Morphosyntax was deficient at the first evaluation and remained impaired later on. However, results on the definitions test were within the normal range at the last evaluation.

Receptive language skills were consistently better than expressive skills. At the last evaluation, receptive language was nearly normal. Auditory discrimination remained excellent. Passive vocabulary was deficient at the first evaluation and slightly improved but nevertheless weak at the second evaluation; it developed favourably starting at 5 years of age and was within the normal range at the last evaluation. Syntactic comprehension was tested after the patient reached 6 years of age; the results were within the normal range until 8 years of age and weak at the last evaluation 1 year later.

Metaphonological skills, necessary for learning written language, were investigated only at the last two evaluations (see Table 7). Syllabic reversal was excellent on both occasions. Phonemic division and consonant subtraction were weak at 8 years of age and within the normal range at 9 years of age.

Overall, whereas most language test results were weak or deficient at the first evaluation, all language skills except morphosyntax were within the normal range at the last evaluation. Thus, the patient's oral-language performance was good at last followup, except regarding morphosyntax. Moreover, he experienced major difficulties with written language: thus, at 8 years of age he was able to recognize only about 20 letters and to read consonant-vowel syllables made up of those letters. He confused letters that have similar shapes. His writing skills were weak. At last followup, his academic skills were at the level of the second grade of primary school.

## 4. Discussion

This paper reports on the prospective 8-year followup, including repeated motor and cognitive testing, of a patient who sustained a bifrontal brain injury at 19 months of age. To our knowledge, this is the first such case report. Although clinical and ecological observations showed steady improvement, severe impairments persisted in important areas such as the IQ, psychomotor skills, and executive functions. Moreover, executive function performance worsened over time.

Previous reports described either adulthood outcomes after bifrontal injury in childhood or childhood outcomes after unilateral frontal injury. Price et al. [27] reported outcomes in 2 patients who sustained bifrontal injuries in childhood and who were evaluated at 28 and 24 years of age, respectively. The main injury was located bilaterally in the frontal lobes. Both patients displayed severe behavioural disorders, and their social and moral development was arrested at an immature stage. Performance was satisfactory in the areas of language, memory, and visuospatial skills used during daily activities. Proof was not obtained that the brain damage in these 2 patients was strictly confined to the prefrontal areas. In 1947, Ackerly and Benton described the case of a 35-year-old man who sustained a bifrontal brain injury at about 3 years of age [28]. His intelligence was described as normal (Stanford-Binet IQ, 92). However, he had behavioural abnormalities with immaturity, inability to learn from experience, lack of drive and curiosity, and irritability when restricted. He failed to benefit from the treatments used and 15 years later he continued to display impulsiveness and inappropriate sexual behaviour [28]. Another patient was evaluated 26 years after she sustained a focal injury to her left frontal lobe at 7 years of age [30]. Although MRI showed a lesion strictly confined to the left prefrontal cortex and underlying white matter, a xenon cerebral blood flow study showed low flow in both frontal regions. Her performance was within the average range for global intelligence, language, memory, and visual perception. However, she exhibited impairments in sustained attention and executive functions.

Because all these patients were examined as adults, the relation between the early frontal damage and the cognitive or behavioural deficits remained unclear. A few prospective case studies were reported more recently. In 1992, Marlowe described how a small injury to the right frontal lobe sustained at 3 years of age disrupted the acquisition of executive and emotional control over the next 3 years, interfering with the development of adaptive behaviours, executive control, emotional regulation, and personality [59]. The patient performed very well on familiar tasks, displaying age-appropriate planning and self-maintenance. On unfamiliar tasks, however, he tended to loose the plan and failed to inhibit maladaptive behaviour. Over the 3-year follow-up, his intelligence scores remained normal on the WPPSI and subsequently on the WISC tests, but he was slow in processing verbal information, exhibited difficulties with visuospatial organisation and productions, and had problems maintaining a mental set. He was able to benefit from experience but at a slower rate than expected. His level of dysfunction increased with the complexity of the environmental demands. Eslinger et al. described the pattern of recovery in a 15-year-old boy who sustained extensive damage to the right dorsolateral prefrontal cortex at age 7 related to rupture and surgical treatment of a deep arteriovenous malformation. Follow-up evaluations 4 and 8 years after surgery showed resolution of left hemispatial neglect and other visuospatial impairments in working memory, design fluency, and planning and organisation. After 8 years, however, he had an acquired form of attention-deficit disorder with impulsivity [32, 60].

Intellectual stagnation over time is of major concern. We recently reported alarming data about the intellectual development of children after severe brain injury in early life [61]: a retrospective review of 50 children showed intellectual deficiencies in 52% of cases and deterioration of intellectual abilities over time. In patients with congenital hemiplegia, intellectual development was nearly normal until 6 to 8 years of age and abnormally slow thereafter [62]. This time-course is at variance with the widespread belief that cortical plasticity in younger patients consistently leads to functionally beneficial reorganization within the brain cortex. Subcortical lesions may make a major contribution to the deterioration over time. The vulnerability of the very young brain may be related to a combination of factors [20, 63, 64] including vulnerability of rapidly emerging skills, vulnerability of established skills, and alterations in recovery or acquisition of skills. Our results are consistent with the hypothesis that brain injury sustained at a very young age is associated with disruptions of cognitive development, especially in the event of bifrontal damage. Late posttraumatic epilepsy developed in our patient 2 years after the injury. He experienced only two brief partial seizures (less than 1 minute) and received carbamazepine for 3 years. Interictal EEGs remained normal. Late posttraumatic epilepsy and carbamazepine therapy may have contributed moderately to the poor neurodevelopmental outcome.

During the 8-year followup after the injury, our patient received one-on-one motor and speech rehabilitation therapy. However, improvements in motor function were slow, and impairments in visuospatial functions and graphic skills persisted. Over time, his intellectual and motor quotients remained within the same range.

The language assessments produced interesting data in our patient. Most areas were impaired at the first evaluation, including phonology, morphosyntax, and lexical processing, for both expressive and receptive language. At the last evaluation, in contrast, only morphosyntax remained deficient. The morphosyntax deficiency may be ascribable to a working memory deficit, as morphosyntax tests involve the manipulation of verbal information. He continued to experience major difficulties with written language: at 8 years of age, he could recognize only 20 letters and read only consonant-vowel syllables made up of those letters; in addition, he confused letters of similar shape. These last difficulties may be ascribable to the deficiency in visual selective attention, corresponding to a more basic deficit [65]. Language development has been described by Rapin [66, 67]. Ewing-Cobbs and Barnes reported that diffuse head injury in young children adversely affected language development, most notably in the lexical and discourse areas [68]. Our results are in agreement with these findings.

Tests for attention showed a persistent deficit in visual selective attention, which was not addressed by remedial therapy. The last two evaluations showed the emergence of difficulties with inhibition. In contrast, symptoms indicating deficient inhibition (e.g., distractibility and restlessness) were reported from the beginning, and some of them resolved over the years. Flexibility was impaired at the next-to-last evaluation but normal at the last evaluation, although clinical observation continued to show perseverative behaviours. The emergence of executive difficulties during the evaluations was consistently delayed compared to the behavioural observations: thus, the test abnormalities did not coincide with the difficulties reported in everyday life. In addition, some abnormalities resolved from one evaluation to the next yet remained present upon clinical observation. These data raise questions about test sensitivity, their ecological relevance, and the possible impact of learning effects. Impairments in planning and organisation were detected only at the last evaluation, whereas several WISC-III subtests (e.g., picture arrangement, described as sensitive to frontal damage and disordered executive function by Van der Linden et al. [69]) were already impaired during previous evaluations. Secondary attention-deficit-hyperactivity-disorder was described in a study of children who were 5 to 15 years of age at brain injury [70]. Executive functions in children who sustained moderate to severe TBI before 6 years of age scored significantly lower than controls on working memory and inhibitory control [71].

We consider that clinical attentional or executive deficits result from early damage of anatomo-functional frontal system, but language, psycho-motor, and visuospatial skills may result from interaction with executive functioning or directly from more diffuse lesions.

Repeated evaluations of working memory showed no progress in storage capacities for tests that are not susceptible to learning effects (digit span and block tapping test). At the last evaluation, storage in the phonological loop and visuospatial pad tests was deficient overall. Remedial treatment for the attentional and executive impairments was not given, for practical reasons.

Regarding behaviour, our patient is the first to be described as exhibiting hyperactivity and difficulties with social integration. Over the years, his hyperactivity decreased, and at last follow-up he was almost too quiet and polite. He experienced persistent difficulties in relationships with his peers, as a result of his interests being appropriate for a younger age group and of his reserved behaviour.

Enrolment in a special-education program became necessary when he was 7 years of age. The program allowed him to substantially improve his language skills. The school evaluations showed evidence of learning, albeit at a slower pace compared to other children. Thus, at last followup, he was 2 grades behind in French and arithmetic, although he had none of the typical signs of dyslexia or dyscalculia.

Our data from a patient with early bifrontal brain damage highlight the importance of long-term followup, as the difficulties change from year to year, in keeping with results described by Eslinger et al. [32, 60]. Some impairments resolved over the years, whereas new impairments emerged. Despite the limitations of the test methods (limited sensitivity, failure to assess ecological factors, and possible impact of learning effects), and despite the limitations of frontal tests (tests are executive only during the first test), our case report also emphasizes the need for detailed assessments. Thus, daily functioning was globally normal in our patient, with an environment adapted to his needs (remedial therapy and special education). This may change over time, because behavioural difficulties may emerge in response to increasing environmental demands, as described by Marlowe [59] and in studies of adults [27–29]. To better understand the cognitive outcomes of early bifrontal early injuries, prolonged in-depth followup is essential.

## Figures and Tables

**Table 1:** Intellectual evaluations (Wechsler scales).

	4 years 10 months	5 years 9 months	6 years 7 months	8 years 3 months	9 years 8 months
	WPPSI-R	WPPSI-R	WISC-III	WISC-III	WISC-IV
Verbal IQ (or verbal comprehension for WISC-IV)	69	76	71	80	84
Performance IQ (or perceptual reasoning for WISC-IV)	76	84	76	73	77
Full-scale IQ	72	78	69	73	73

*Mean 100, standard-deviation 15.

**Table 2:** Attentional evaluations.

	6 years 7 months		8 years 3 months		9 years 8 months	
	Z Score	Raw Score	Z Score	Raw Score	Z Score	Raw Score
*Visual selective attention*						
Cats crossing (NEPSY)						
Times (sec.)	+0.1	63	+0.6	35	+0.3	39
Omissions	−0.1	1	−0.5	1	+0.4	0
Commissions	+0.1	0	+0.1	0	+0.2	0
Faces crossing (NEPSY)						
Times (sec.)	−0.7	180	−0.02	140	+0.7	107
Omissions	+0.4	4	−1.4	7	−2.3	8
Commissions	−0.4	15	−2.2	12	−0.4	4
Visual attention task (TAP)						
Mean reaction times	+2.3	879	−0.2	1274	−0.2	1255
Omissions	+1.9	4	−1.5	9	−0.4	5
Commissions	−15.4	32	−3.0	7	+0.8	0

*Sustained Attention*						
10-minute Zazzo						
Speed	—	—	−1.0	360	−1.0	439
Accuracy	—	—	−3.0	41.5	−0.5	14.5

*Divided Attention*						
Divided Attention Task (TAP)						
Reaction times (msec.)	—	—	−0.5	975	−0.03	946
Omissions	—	—	−1.7	13	−0.9	8
Commissions	—	—	+0.4	1	+0.9	0

**Table 3:** Executive function evaluations.

	6 years 7 months		8 years 3 months		9 years 8 months	
	Percentile	Raw score	Percentile	Raw score	Percentile	Raw score
*Inhibition*						
Statue (NEPSY)	26–75	25	11–25	25	26–75	27
Knock and Tap (NEPSY)	26–75	27	11–25	22	26–75	29
Go/No-Go (TAP)						
Median reaction times (msec.)	—	—	97	358	88	404
Omissions	—	—	3	4	3	4
Commissions	—	—	3	9	4	8
Go/No-Go (KITAP)						
Median Reaction Times (msec.)	—	—	>100	331	66	442
Omissions	—	—	10	2	42	3
Commissions	—	—	5	7	>34	0
Incompatibility (TAP)						
Median reaction times (msec.)	—	—	—	—	98	338
Commissions	—	—	—	—	<1	31

	Z score	Raw score	Z Score	Raw Score	Z Score	Raw Score

Fruit Stroop Task						
Naming times (sec.)	−2.5	65	−0.4	36	−0.2	35
Naming errors	−0.7	2	−4.3	2	−4.3	2
Interference times (sec.)	−0.8	97	−2.8	82	−2.9	83
Interference errors	−0.6	4	−4.5	7	−5.3	8

*Flexibility *						
Flexibility Task (TAP)						
Mean reaction time (msec.)	−0.8	2694	−0.5	1570	+0.03	1363
Hits	−0.8	39	−2.1	30	−0.4	42
Errors	−1.1	11	−2.1	15	−0.3	8
Verbal fluency (NEPSY)						
Animal	+0.3	11	+0.9	15	+1.3	19
Beverages and foods	−1.5	4	−0.01	12	−0.4	11
Design fluency (NEPSY)						
Structured array	−0.9	4	+0.1	11	+0.4	13
Random array	−0.8	5	−0.6	9	−0.02	12

*Planning*						
Tower of London						
First trials	—	—	+0.6	7	+2.0	9
Total trials	—	—	+0.9	19	+2.1	15
Planning times (sec.)	—	—	+0.8	4.5	−1.6	7.4
Execution times (sec.)	—	—	+0.3	5.6	−1.5	8

**Table 4:** Working memory evaluations.

	4 years 10 months		5 years 9 months		6 years 7 month		8 years 3 months		9 years 8 months	
	Z-score	Raw score	Z-score	Raw score	Z-score	Raw score	Z-score	Raw score	Z-score	Raw score
*Phonological loop*										
Digit span	−1.75	2	−0.7	3	−0.8	3	−2.9	3	−2.0	3
Reverse digit span	—	—	—	—			−2.5	2	−0.4	3
Word set (K.ABC)	−2.3	3	−1.6	5	−1.3	6	−1.3	6	—	—

*Visuospatial pad*										
Block Tapping Test	−0.7	3	−1.9	2	−2.5	2	−1.9	3	—	—
Hand movements (K.ABC)	−1.6	5	−0.6	8	+0.3	11	−1.6	5	—	—
Spatial memory (K.ABC)	—	—	−0.6	8	−0.3	9	−0.6	8	—	—

**Table 5:** Psychomotor evaluations.

	3 years 9 months	4 years 10 months	5 years 9 months	8 years 3 months	9 years 8 months
*Oseretsky Scale*					
Basis age	2 years	3 years	3 years	4 years	4 years
Motor age	2.8 years	3.6 years	4.4 years	5.10 years	6.8 years
Motor quotient	73	74	76	71	70

**Table 6:** Language assessments from 3 to 5 years of age.

	3 years 9 months		4 years 10 months		5 years 9 months	
	Percentile	Raw Score	Percentile	Raw Score	Percentile	Raw Score
*Expression*						
Praxis: BEPL (Pra)	85	93.3%	50	86.7%	—	—
Semantic fluency: Mc Carthy	—	—	—	—	33	13 words
Phonology						
BEPL PHO1	12	61%	11	79%	<1	73.6%
BEPL PHO2	7	50.6%	15	77%	<1	69.5%
Repetition						
Syllables						
BEPL Art 1	1-2	62.5%	9*	75%	1*	62.5%
BEPL Art 2	—	—	23*	73.3%	5*	60%
Sentences						
BEPL RPH1	—	—	16*	20/26	60*	24/26
BEPL RPH2	—	—	5*	24/40	35*	31/40
Morphosyntax						
TCG	<1	4/52	2	14/52	<1	19/52
TVAP definitions	—	—	3	13/60	—	—

*Comprehension*						
Auditory discrimination						
BEPL Gno	45	50%	82	100%	—	—
TVAP 3–5	<1	18/60	6	41/60	25	50/60
EVIP (Peabody)	3	8/170	—	—		

Reynell	Developmental age		Developmental age		Developmental age	
	3 years	39	3,11 years	50	5 years	59

*Normative data are available for children up to 4 years 3 months of age.

**Table 7:** Language assessments from 6 to 9 years of age.

	6 years 7 months		8 years 3 months		9 years 6 months	
	Percentile	Raw Score	Percentile	Raw Score	Percentile	Raw Score
*Expression*						
Praxia: HENIN	—	—	50	46/62	—	—
Semantic Fluency: Mc Carthy	79	21 words	73	27 words	50	20 words
Phonemic Fluency: L2MA	—	—	4	6 words	50	13 words
Vocabulary						
EEL LX2/NEEL voc 1	<1	19%	4	47/72	68	64/72
EEL LX3/NEEL voc 2	1	29%	5	32/42	33	36/42
L2MA	—	—	10	9/25	23	13/25
Phonology						
EEL Dex	11	90.3%	—	—	—	—
NEEL monosyllabic words	—	—	<1	24/28	62	28/28
NEEL polysyllabic words	—	—	<1	44/50	61	50/50
Repetition						
Words						
EEL Rep	<1	84.8%	—	—	—	
NEEL monosyllabic words	—	—	<1	26/28	57	28/28
NEEL polysyllabic words	—	—	54	50/50	54	50/50
Sentences						
EEL PH1	16	50%	—	—	—	—
EEL PH2	4	60%	—	—	—	—
NEEL B1 Syntax	—	—	<1	0/2	64	2/2
NEEL B1 Numbers of words	—	—	<1	19/31	9	24/31
NEEL B2 Syntax	—	—	50	0/1	54	1/1
NEEL B2 Numbers of words	—	—	19	13/25	41	17/25
Non words						
BELEC CV	15	13/20	38	15/20	50	16/20
BELEC CV Span (syllables)	51	5	58	5	58	5
BELEC CCV	—	—	37	9/20	47	10/20
BELEC CCV Span (syllables)	—	—	37	3	30	3
Morphosyntax						
TCG	3	23/52	5	32/52	5	38/52
TVAP definitions	16	29/60	2	25/60	37	40/60

*Comprehension*						
Auditory discrimination						
Words						
EDP 4–8	>80	32/32	>80	32/32	>80	32/32
TVAP 5–8	35	52/60	16	47/60	63	55/60
EVIP (Peabody)	25	57/170	45	80/170	60	105/170
Sentences						
O52 (Khomsi)	50	46/52	50	49/52	—	—
ECOSSE	17	22 errors	20	12 errors	6	14 errors

*Metaphonology*						
Syllabic reversal (BELEC)	—	—	90	10/10	90	10/10
Phonemic reversal (BELEC)	—	—	3	5/10	90	10/10
Consonant subtraction (BELEC)	—	—	<10	0/10	50	9/10
